# *Citrus limon* from Tunisia: Phytochemical and Physicochemical Properties and Biological Activities

**DOI:** 10.1155/2018/6251546

**Published:** 2018-01-15

**Authors:** Mohamed Makni, Raoua Jemai, Walid Kriaa, Yassine Chtourou, Hamadi Fetoui

**Affiliations:** ^1^Laboratory of Toxicology, Environmental Microbiology and Health, Science Faculty of Sfax, University of Sfax, Sfax, Tunisia; ^2^Laboratory of Plant Biotechnology, Faculty of Sciences of Sfax, University of Sfax, Sfax, Tunisia

## Abstract

Natural plant extracts contain a variety of phenolic compounds which are assigned various biological activities. Our work aims to make a quantitative and qualitative characterization of the Zest (ZL) and the Flesh (FL) of lemon* (Citrus limon)*, to valorize the pharmacological uses of lemon, by evaluating* in vitro* activities (DPPH, free radical scavenging and reducing power). The antibacterial, antifungal, and antiproliferative activities were sought in the ability of* Citrus limon* extracts to protect DNA and protein. We found that the ZL contains high amounts of phenolics responsible for the important antioxidant properties of the extract. However, the FL is richer in flavonoids than the ZL. The FL extract was also found to be more effective than the ZL in protecting plasmid DNA against the strand breakage induced by hydroxyl radicals. We also concluded that the FL extract exhibited potent antibacterial activity unlike ZL. Analysis by LC/MS-MS identified 6 compounds (Caffeoyl N-Tryptophan, Hydroxycinnamoyl-Oglucoside acid, Vicenin 2, Eriocitrin, Kaempferol-3-O- rutinoside, and Quercetin-3-rutinoside). These preliminary results showed that* Citrus limon* has antibacterial and antioxidant activity* in vitro*. It would be interesting to conduct further studies to evaluate the* in vivo* potential in an animal model.

## 1. Introduction

In recent years, many diseases have appeared and are mainly due to “oxidative stress” which is the result of an imbalance between the formations or not of prooxidants [1]. Indeed, reactive oxygen species (ROS) are reactive molecules due to the presence of unpaired electrons such as superoxide anion radicals (O_2_^•−^), hydroxyl radicals (OH^•^), hydroperoxyl radicals (HOO^•^), peroxyl (ROO^•^), and also nonradical species such as hydrogen peroxide (H_2_O_2_), ozone (O_3_), and singlet oxygen (^1^O^•^_2_) [2].

Oxidative stress is caused by the presence of free radicals that upset stability by electronic pairing with several biological macromolecules such as proteins, lipids, and DNA and cause significant damage to the basic structures of the body (proteins, lipid, and DNA) [1, 3]. Evidences that ROS accumulation in biological systems causes oxidative tissue damage and affects cellular integrity and function are tangible. Oxidative damage caused by ROS has often been the origin of the pathogenesis of several diseases such as aging, arthritis, cancer, inflammation, and heart disease [4].

Lemon is among the most important crops in the world, with an annual production of about 123 million tons in 2010. Lemon (*Citrus limon* L.) occupies the third most important* Citrus* species after orange and mandarin world production by 4.200.000 metric tons [5].

Lemon (*Citrus limon* L.) is a main element of the Tunisian economy. In fact, lemon and lime production reached nearly 27.000 tons in 2005 [6]. The genus* Citrus* includes several important species worldwide, oranges by 56%; tangerines and clementines: 17%; lemons and limes: 11%; and finally grapefruit: 6% of the total [7].

Lemon is very rich in important natural compounds, including citric acid, ascorbic acid, minerals, flavonoids, and essential oils. Therefore, although the new* Citrus* cultivars have been mainly developed for fresh consumption, the particular characteristics such as their phenolic compound and in particular the flavonoids contents have led to their use in new fields such as pharmacology and food technology [8].

Citrus fruits are mainly used in food industries for the production of fresh juices. Thanks to their important composition in bioactive molecules (natural antioxidants, phenolic acids, and flavonoids), peels, the main fraction of* Citrus* waste which represent approximately half of the mass of the fruit, have been widely studied [2].

Therefore, it is of great interest to screen these plants in order to validate their use in food and medicine and to reveal the active ingredient by characterizing their constituents. The aim of this study was to investigate the* in vitro* antioxidant activities of extracts from the peel (ZL) and the Flesh (FL) of* Citrus limon*. Studies included DPPH free radical scavenging and reducing power. In addition, a determination of the antibacterial and antiproliferative assay was sought. Thus, we made tests of DNA damage and protein to assess the protection ability of extracts.

## 2. Materials and Methods

### 2.1. Samples

In his study,* Citrus* fruits* (Citrus limon)* were collected and harvested in mature period: stage yellow color in April 2013 from Sfax, Tunisia. Fruits of lemon cultivar Beldi were yellow-colored and oblate spheroids. The investigation was carried out at the mature stage. Citrus fruits were divided into two parts: the Zest of lemon (ZL) and the Flesh of lemon (FL). Zest is the outer colored portion of the* Citrus* peel and the Flesh is the fruit peels including flavedo (epicarp) and albedo (mesocarp) layers.

### 2.2. Preparation of the Hydroethanol Extracts

The two extracts ZL and FL were prepared. In brief, 100 g of each part of the plant (ZL and FL) was extracted by 300 ml of ethanol-water (7 : 3, v/v) with shaking for 24 h at a rotational speed of 200 rpm. After 24 h, the ethanol-soluble fraction was filtered and concentrated under reduced pressure at 45°C using a rotary evaporator. Finally, the extract was lyophilized and kept in the dark at 4°C. Extraction yields of ZL and FL were 10.64% and 14.33%, respectively.

### 2.3. Determination of Phenolic Compound

#### 2.3.1. Total Phenolic Content (TPC)

The Folin-Ciocalteu assay, adapted from Zou et al. (2011) [9] with minor modifications was used for the determination of total phenolics present in the* Citrus* fruit extracts. Briefly, 10 *μ*l of appropriately diluted extracts or standard gallic acid solutions was mixed with 20 *μ*l of a Folin-Ciocalteu reagent solution in a 96-well plate and mixed gently. After five minutes, 30 *μ*l of freshly prepared 20% sodium carbonate was added followed by 158 *μ*l of distilled water. The reaction mixture was kept in dark for 2 h and the absorbance of the blue coloration formed was measured at 765 nm against the blank solution, which was prepared by the same procedure described above except the extract solution was substituted by 10 *μ*l of ethanol, using the microplate reader. The TPC was expressed as mg gallic acid equivalent (mg GAE/g).

#### 2.3.2. Determination of Total Flavonoid Content (TFC)

Total flavonoids in the extracts were determined using a slightly modified colorimetric method described previously by Zou et al. (2011) [9]. A 30 *μ*l aliquot of appropriately diluted sample solution was mixed with 180 *μ*l of distilled water in a 96 well plate, and subsequently 10 *μ*l of a 5% aqueous NaNO_2_ solution was added. After six minutes, 20 *μ*l of a 10% of aluminum chloride solution was added and allowed to stand for six minutes; then 60 *μ*l of 4% NaOH solution was added to the mixture and stood for another 15 min. Absorbance of the mixture was determined at 510 nm versus a prepared water blank using a Multiskan Spectrum microplate reader. Total flavonoids were calculated with respect to quercetin standard compound (12.5, 25, 50, 75, and 100 *μ*g/ml). All values were expressed as milligrams of quercetin equivalents per 1 g sample (mg QEeq/g sample).

#### 2.3.3. Determination of Flavonol Content

The flavonol content was measured using a colorimetric assay adapted from Yermakov et al. (1987) [10] with slight modifications. The rutin calibration curve was prepared in a well of 96-well plate by mixing 40 *μ*l of various concentrations of ethanolic solutions of rutin with 40 *μ*l (20 mg/ml) aluminum trichloride and 120 *μ*l (50 mg/ml) sodium acetate. The absorbance at 440 nm was read after 2.5 h. The same procedure was used for 40 *μ*l of plant extract instead of rutin solution. All determinations were carried out in triplicate. The flavonol content was calculated using a standard curve obtained from various concentrations of rutin (0–50 *μ*g/ml). All values were expressed as milligrams of rutin equivalents per 1 g sample (mg REeq/g sample).

#### 2.3.4. Determination of Condensed Tannin Content (CTC)

The CTC in the extracts and its fractions was determined using the modified vanillin assay [9]. Ten *μ*l of appropriately diluted sample solution was mixed with 120 *μ*l of 4% vanillin solution (in methanol) in a well of 96-well plate, and then 60 *μ*l of concentrate HCl was added and mixed. After 15 min, the absorbance of the mixture was determined at 500 nm against a blank solution, which was prepared by the same procedure described above except the extract solution was substituted by 10 *μ*l of water. Different concentrations of catechin ranging from 25 to 300 *μ*g/ml were used as standard compound for the quantification of total condensed tannins. All values were expressed as milligrams of catechin equivalents per 1 g sample (mg CEeq/g) [9].

### 2.4. Liquid Chromatographic and Spectrophotometric Mass Analysis

LC-MS/MS analyses were performed on the apparatus consisting of elements following Thermo LTQ HPLC System, LC system equipped with a quaternary pump, auto-sampler, and a UV diode array detector and mass spectrometer Agilent Triple Quadrupole Ion Trap XCT MSD: spectrometer mass fitted with an electrospray ionization interface, controlled by software Analyst (version 1.3.1).

The extracts were injected onto a HPLC column Zorbax C-18 300 Å (2.1 × 150 mm). The separation was conducted at ambient temperature with a mobile phase consisting of two water 0.1% formic acid solvent (A) and acetonitrile (B) in the following conditions: 5% B for 35 min, followed by a 11 min linear gradient from 5 to 100% B, then 100% B for 4 min, and, finally, back to initial conditions (5% B) in two minutes to balance the column before reinjection. For all analyses, the solvents used were HPLC grade; the speed was set at 200 *μ*l/min. The injection volume was 5 *μ*l.

### 2.5. Antioxidant Capacity Assays

#### 2.5.1. 2,2-Diphenyl-1-picrylhydrazyl (DPPH) Free Radical Scavenging Activity Assay

The antioxidant activity of the extracts was firstly evaluated by monitoring its ability in quenching the stable free radical DPPH. The radical scavenging activity of the extracts and fractions against DPPH free radicals was measured using the method of Clarke et al. (2013) [11] slightly modified as follows: 20 *μ*l of appropriately diluted samples or Vitamin C solutions (10, 50, 100, 500, and 1000 *μ*g/ml) was added to 190 *μ*l of DPPH solution (100 *μ*M) in a well of 96-well plate. The mixture was shaken vigorously and allowed to reach a steady state at room temperature for 30 min. Discoloration of DPPH was determined by measuring the absorbance at 517 nm with a Beckman spectrophotometer. All determination was carried out in triplicate. Ascorbic acid was used as a positive control. The DPPH radical scavenging activity was calculated according to the following equation:

(1) $$\begin{array}{c} \text{Scavenging rate}=\left[\;1-\frac{\left(A_{1}-A_{2}\right)}{A_{0}}\;\right]\times 100\%, \end{array}$$

where *A*_0_ was the absorbance of the control (blank, without extract), *A*_1_ the absorbance in the presence of the extract, and *A*_2_ the absorbance without DPPH.

#### 2.5.2. Reducing Power Assay

The Fe^3+^ reducing power of the extracts was determined by the method of Verma and Banerjee (2010) [12] with slight modifications. The ethanolic extracts, ascorbic acid, were used at different concentrations (7.8, 15.6, 31.25, 62.5, 125, 250, and 500 *μ*g/ml). One milliliter of each sample was mixed with phosphate buffer (2.5 mL, 0.2 mol·L^−1^, pH 6.6) and potassium ferricyanide [K_3_Fe (CN)_6_] (2.5 mL, 30 mmol·L^−1^) followed by incubating at 50°C in a water bath for 20 min. The reaction was stopped by adding 2.5 ml of trichloroacetic acid (TCA) solution (10%) and then centrifuged at 3000 r/min for 10 min. The supernatant (100 *μ*l) was mixed with distilled water (100 *μ*l) and FeCl_3_ (20 *μ*l, 0.1%), in a well of 96-well plate, and the absorbance was measured at 700 nm as the reducing power in a spectrophotometer. Higher absorbance of the reaction mixture indicated greater reducing power.

### 2.6. Determination of the Antibacterial Activity

#### 2.6.1. Microorganisms for Study

A total of nineteen pathogenic microbial cultures including ATCC strains of bacterial and fungal origin were taken for this study. Eleven of the bacteria and eight fungal strains were isolated from clinical specimen obtained from patient samples and identified by standard laboratory protocol.

Gram Positive* Streptococcus agalactiae B, Streptococcus D, Enterococcus, Staphylococcus aureus*, Gram Negative* Escherichia coli, Citrobacter koseri, Acinetobacter baumannii, Proteus mirabilis, Klebsiella pneumoniae, Salmonella enterica, Pseudomonas aeruginosa, *and Fungal* Aspergillus niger, Penicillium *spp.*, Microsporum canis, Trichophyton violaceum, Cryptococcus neoformans, Candida albicans, Candida tropicalis, and Candida glabrata *were considered.

#### 2.6.2. Antimicrobial Activity

The plant extracts were dissolved in dimethyl sulfoxide (DMSO) at a concentration of 10 mg/mL and tested for antibacterial activity by the agar well diffusion assay. The bacterial culture in Muller Hinton broth was adjusted to the final inoculum density of 1 × 10^7^ CFU/mL (by 0.5 McFarland standards) on molten Muller Hinton agar (MHA) plates. After solidification, wells (diameter 9 mm) were made with a sterile borer in the inoculated MHA plates. About 100 *μ*L solution containing 1 mg of each extract was dispensed in the wells, while DMSO was also tested as the vehicle control. Penicillin G, streptomycin, and gentamicin were the standard drugs used as positive controls in this assay. Antibacterial activity was expressed as the diameters of inhibition zones produced around each well by the plant extracts and antibiotics and was measured after 24 h of incubation at 37°C. Each test was conducted in triplicate to confirm the reproducibility of the observed data [13].

#### 2.6.3. Antifungal Activity

The crude plant extracts as described above were screened for antifungal activity. Fungal culture in Sabouraud dextrose broth containing an inoculum density of 0.5 McFarland (1 × 10^8^ CFU/mL) was diluted at 1 : 10 ratio in SDA plate to obtain the final inoculum concentration of 1 × 10^7^ CFU/mL. Wells (diameter 6 mm) were punched on solidified SDA plates and 100 *μ*L solution containing 1 mg of each extract was dispensed in the wells. Amphotericin-B was used as a standard drug for antifungal assay, and DMSO was tested as the vehicle control. The diameter of the inhibition zone was measured after 24 h of incubation at 35°C. Antifungal activity was expressed as diameters of inhibition zones produced by the plant extracts and antifungal agent. Each test was conducted in triplicate and the reproducibility of the observations was confirmed [13].

### 2.7. Determination of the Antiproliferative Activity

#### 2.7.1. Cell Line: Strain B95-8 (ATCC: VR-1492)

This is a lymphoid line producing virions Epstein-Barr transformants. It was obtained from lymphocytes B of marmoset and irradiated lines from patients with infectious mononucleosis. A fraction of 1–3% of B95-8 cells enters spontaneously in a viral lytic cycle [14]. Original laboratory is laboratory of cell culture, Habib Thameur Hospital of Tunis.

#### 2.7.2. Culture Medium

The culture medium RPMI 1640 (Rosewell Park Memorial Institute) (Gibco) was used for the culture of lymphoblastoid cell line: B95-8. The medium was supplemented with 2 g/l sodium bicarbonate (HCO_3_ Na). After adjusting the pH to 7.2 with 1 N HCl, the mixture was filtered through a filter of 0.22 microns and then supplemented with 10% fetal bovine serum (FBS) (Gibco), gentamycin 1%, and L-glutamine 2 mM.

#### 2.7.3. Cell Culture

*(i) Maintenance Culture Cells.* All cell lines were cultured in culture flasks (Iwaki) of 25 or 75 cm^2^. Transplanting cells was carried out every 3–5 days.

Cells that have reached the saturation concentration were centrifuged for 10 min at 1000 rpm and then suspended in 2 ml of RPMI medium supplemented with 10% fetal bovine serum (FBS). After counting in the presence of trypan blue, the cells were placed in culture at a concentration of 2 · 10^5^ cells/ml [15].

*(ii) Trypan Blue Exclusion Test (Cell Count). *The test of trypan blue exclusion (Sigma) is based on the evaluation of the integrity cell membrane. It is a specific technique for cell counts and assessment of cell death. It consists of an optical microscope to count the number of cells present in a given volume of cell suspension. Counting was performed on a Malassez cell. 20 *μ*l of cell suspension was diluted with 20 *μ*l of trypan blue. After mixing, a small volume was set in the cell count for Malassez. The concentration of the number of cells per ml was given by the following formula:

(2) $$\begin{array}{c} \text{N}=n\times 10\times \text{dilution factor}\times 1000 \end{array}$$

where *N* is number of cells per ml.

*(iii) Cellular Cytotoxicity Test (MTT Assay).* The MTT (3 Bromide (4.5-dimethylthiazol-2-yl)-2.5-diphenyltetrazolium bromide) (Sigma, Germany) is initially yellow and the substrate is a mitochondrial enzyme succinate dehydrogenase. The latter is capable of cleaving certain covalent bonds of MTT, which transforms it into formazan salt (purple salt), insoluble in aqueous media. This reaction can be monitored quantitatively by spectrophotometry. The DO at 570 nm reflects the activity of mitochondrial cytochromes. This activity can be considered as an index of cell proliferation [16].

### 2.8. Protein Damage Protection Assay

The effects of the sample on protein oxidation were carried out according the method of Hu et al. (2012) [17] with minor modifications. BSA was oxidized by a H_2_O_2_/Fe^3+^/ascorbic acid system. The reaction mixture (1.0 ml), containing 0.2 ml of sample, 0.2 ml of phosphate buffer (100 mM, pH 7.4), 0.2 ml of BSA (5 mg/ml), 0.2 ml of FeCl_3_ (250 *μ*M), 0.1 ml of H_2_O_2_ (20 mM), and 0.1 ml of ascorbic acid (1 mM), was incubated for 6 h at 37°C. After incubation, the reaction mixture was analyzed by electrophoresis in 10% SDS polyacrylamide gel. The gel was stained with a brilliant blue R staining solution for 2 h, destained, and digitally photographed.

### 2.9. Plasmid DNA Damage Assay

DNA damage and DNA protecting activities of Citrus extracts were prospected on pBR322 plasmid DNA. The plasmid DNA was oxidized with H_2_O_2_ + UV treatment in the presence or absence of extracts of Citrus according to protocols of Jagtap et al., 2011 [18]. In brief, the experiments were performed in a volume of 15 *μ*l in an Eppendorf tube containing 200 ng of pBR322 plasmid DNA. H_2_O_2_ was added to final concentration of 100 mM with or without 10 *μ*l of Citrus extracts. The reaction mixture was exposed to UV irradiation and continued at ambient temperature for 5 min on the surface of UV mini trans-illuminator. After irradiation, the mixture was incubated at room temperature for 15 min. To the mixture, gel loading dye was added and the fragments were separated by electrophoresis. Untreated plasmid DNA was used as a control in each run of gel electrophoresis along with UV and H_2_O_2_ treatments.

### 2.10. Statistical Analysis

Experimental results are expressed as means ± SD. All measurements were replicated three times. The data were analyzed by an analysis of variance (*P* < 0.05) and the means separated by Duncan's multiple range test. The IC50 values were calculated from linear regression analysis.

## 3. Results and Discussion

### 3.1. Total Phenolic, Flavonoid, Flavonol, and Tannin Contents

Total phenol compounds, as determined by Folin-Ciocalteu method, are reported as gallic acid equivalents with reference to standard curve (*y* = 0.003*x*, *R*^2^ = 0.999). The total phenolic contents were usually significantly higher in Zest (*P* ≤ 0.001) with the range of 204.4 ± 9.62 than Flesh with the range of 105.55 ± 4.71 mg gallic acid equivalent/g of extract (Table 1). Polyphenols are the major plant compounds with significant antioxidant activity. The antioxidant activity of these compounds is mainly due to their redox properties [19, 20]. Our results on polyphenol contents were higher than those measured in similar varieties from Iran and Portugal (131 and 87 mg EAG/g extract, resp.) [21, 22]. Indeed, these results indicate that the polyphenol content may be influenced by various factors such as genotypic differences, geographic and climatic conditions, time of harvest, and even cultural practices [23].

de Lourdes Mata Bilbao et al. (2007) [24] showed a rate of polyphenols in Zest of lemon about 3524 mg EAG/100 g of extract, while Guimarães et al. (2010) [22] showed a rate of polyphenols of 87.77 mg EAG/g of extract. This difference probably resulted from the fact that the determination by the Folin-Ciocalteu reagent is not specific to polyphenols, but thousands of compounds may react with the reagent, giving a higher apparent phenolic rate [25, 26]. The phenol content of a plant depends on a number of intrinsic and extrinsic factors [27].

The total flavonoid contents were significantly higher in FC (*P* ≤ 0.01) with the range of 56.16 ± 14.14 with respect to ZC with the range of 27.5 ± 6.88 mg QEeq/g of extract powder with reference to standard curve (*y* = 0.003*x*, *R*^2^ = 0.981) (Table 1). In recent years, particular attention has been given to a specific class of phytochemical antioxidants which are flavonoids. Flavonoids are polyphenolic substances naturally present in almost all plant materials and are prominently ubiquitous in cereals, vegetables, fruit, nuts, wine, tea, beer, and cocoa [28]. These flavonoid compounds have a broad spectrum of chemical and biological activities. Indeed, they are compounds which possess strong antioxidant properties. Their potential ability to capture and chelate metals and ROS depends on chemical structures and the number and position of hydroxyl groups. Flavonoids such as tea catechins show a high activity of the ferrous iron chelate [4]. Comparative studies by Wang et al., (2014) [29] and Guimarães et al. (2010) [30] proved that the extract of lemon has flavonoid contents of the order of 32.7 and 15.96 mg QEeq/g extract, respectively. Flavonoids, one of the most widespread and diverse groups of natural compounds, are probably the most important natural phenolic compounds. Several biological effects* in vitro* and* in vivo* due to the consumption of foods containing flavonoids were demonstrated. Epidemiological studies showed that increased consumption of flavonoids reduces the risk of cardiovascular disease and certain types of cancer [31].

Flavonols are reported as rutin equivalents with reference to standard curve (*y* = 0.002*x*, *R*^2^ = 0.997). The content of flavonols was significantly higher in Zest (*P* ≤ 0.001) with the range of 26.66 ± 7.07 and of the range of 9.16 ± 3.53 mg REeq/g of extract powder (Table 1). We note that the majority of flavonoids for Zest of lemon consist of flavonols. The study of Wang et al. (2014) [29] showed that the majority of flavonols Zest of lemon are quercetin and rutin. In fact, their concentrations are about 0.573 and 0.060 mg mg REeq/g, respectively.

The content of condensed tannin was significantly higher in Zest (*P* ≤ 0.001) with the range of 138.33 ± 35.35 than the Flesh with the range of 26.66 ± 18.92 mg catechin equivalent/g of extract powder with reference to standard curve (*y* = 0.002*x*, *R*^2^ = 0.994) (Table 1). The tannins are secondary compounds of various chemical structures, widely produced in the plant kingdom and generally divided into hydrolysable and condensed tannins. Condensed tannins are found primarily in the walls of seeds and play an important role in the defense system of seeds that are exposed to oxidative damage by many environmental factors such as light, oxygen, free radicals, and metal ions [32].

Following the results of the quantitative characterization, lemon is a promising source of beneficial bioactive compounds for human health through its constituent polyphenols and flavonoids.

### 3.2. Identification of the Phenolic Composition of ZL Extract

The analysis of the ZL extract of* Citrus limon* in liquid chromatography high performance coupled with mass spectrometry (LC-MS/MS) identified compounds which are greater in number of 6 phenolic products (Caffeoyl N-Tryptophan, Hydroxycinnamoyl-Oglucoside acid, Vicenin 2, Eriocitrin, Kaempferol-3-O- rutinoside, and Quercetin-3-rutinoside) as described in Table 2 in order of elution.

The presence of Citrus flavonoids is manifested chiefly in glycoside or aglycone forms [33]. In fact, flavonoids are more abundant in Zest than seeds [34]. Lemon seeds are richer in eriocitrin but poorer in naringin. Meanwhile, the Zest contains important contents of neoeriocitrin, neohesperidin, and naringin and is poor in narirutin [35, 36].

Miyake et al. [37] performed the isolation of two C-glucosyl flavones from lemon fruit: diosmetin 6, 8-di-C-glucoside and diosmetin 6-C-d-glucoside. Moreover, such flavones are found in limes, rather than other kinds of Citrus fruit [34, 38]. Lemon juices were less rich in vicenin-2, and diosmin [39–41]. However, three most abundant flavones were found in lemon Zest: diosmetin 6,8-di-C-glucoside [37], vicenin-2, and diosmin [35].

Rutin and myricetin were most identified in lemon juice [42, 43], but quercetin and kaempferol existed in Zest and juice as well [36, 41, 42]. Hydroxycinnamic acids were also detected in very low concentrations (caffeic, chlorogenic, ferulic, sinapic, and p-coumaric acids) [40, 41, 44, 45].

### 3.3. Antioxidant Activity of* Citrus limon* Extracts

The antioxidant activity cannot be evaluated by only a single method due to the complex nature of phytochemicals. Also, the antioxidant activity determination is reaction-mechanism dependent. Therefore, it is important to employ multiple assays to evaluate the antioxidant activity of plant extract or phytochemicals [9].

#### 3.3.1. The Scavenging Activity for DPPH Radicals

DPPH is a stable organic free radical with a strongest adsorption at 517 nm, the color of which turns from purple to yellow followed by the formation of DPPH upon absorption of hydrogen from an antioxidant [46].

**Figure 1:**
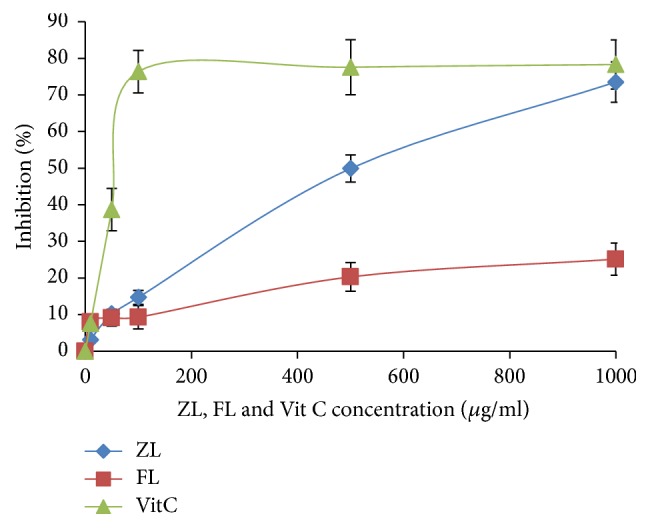
Radical scavenging effect (%) on DPPH (2,2-diphenyl-1- picrylhydrazyl) radicals of ZL and FL extracts of* Citrus limon*. The values are the mean of three determinations ± SD.

DPPH molecules that contain a stable free radical have widely been used to evaluate the radical scavenging ability of antioxidants. The free radical scavenging activities of the two extracts, ZL and FL, were assayed by using DPPH. As shown in Figure 1, both ZL and FL reacted directly with and quenched DPPH radicals to different degrees with increased activities at higher concentrations. At all of the concentrations tested, ZL showed significantly stronger activities than FL. However, at similar concentrations, the scavenging effect of FL was only 20.3% ± 3.9. The IC50 of ZL was about 434.50 *μ*g/ml ± 5.9. To obtain the same IC50 scavenging activity, the concentration needed for FL was 1126990.76 mg/ml ± 9.2, almost 2596 times; although both ZL and FL showed DPPH scavenging activity, ZL was a considerably better DPPH radicals scavenger. The antioxidant potential of extracts was different may be due to the difference in chemical structures of their phenolic compounds, as suggested by previous work as regards the relationship between the chemical structure and antioxidant potential of phenolic compounds by means of the DPPH method [46]. The antioxidant capacity is worth evaluating in three structural groups [47], the first of which is the B-ring* ortho*dihydroxy (catechol) structure. This structure favors the stability to aroxyl radicals, possibly thanks to hydrogen bonding. It also leads to electron dislocation. The 2, 3-double bond conjugated with a 4-oxo function is the second structure responsible for B-ring electron dislocation. Finally, we mention hydroxyl groups. Evidently, a combination of these chemical and structural elements is responsible for the flavonoid antioxidant capacity. An example is the presence or absence of glycosides or aglycones and the amount and position of eventually esterified hydroxyls [48, 49].

At position 3 in flavanones and flavones, the lack of a hydroxyl group affects their antioxidant ability. However, at 2 and 3 the double bond increases the structure reactivity. Thus, apigenin is denoted as a moderate antioxidant compound, while naringenin is not active against the superoxide ion.

#### 3.3.2. The Reducing Power

The reducing power has widely been used as a significant marker of the antioxidant activity. In this assay, the yellow color of the solution acquired various green and blue shades due to the reducing power of compounds. Antioxidants lead to the Fe^3+^ reduction in the presence of a ferricyanide complex to the ferrous (Fe^2+^) form through a one electron donation [50].

**Figure 2:**
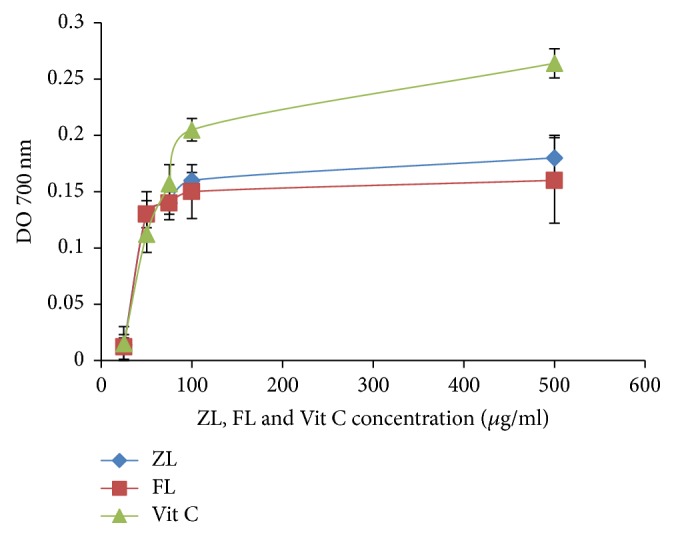
Reducing power of ZL and FL extracts of* Citrus limon*, as measured by changes in DO at 700 nm. The values are the mean of three determinations ± SD.

As shown in Figure 2, we obtained a significant value of reducing power in both ZL and FL extracts. Furthermore, the data indicated a concentration-dependent mode for the reducing powers of both extracts. In addition, the latter also increased in parallel with concentrations. This is due to their richness in bioactive molecules that act as antioxidants. The considered extracts' relatively strong reducing power was noticeable. However, the ZL extract was found to have slightly higher reducing activity than FL. The hydrogen- or electron-donating capacity of these extracts could be the cause behind this phenomenon [51]. Accordingly, relatively higher amounts of reductones could be found in both extracts. Possibly, these reductones could react with free radicals to stabilize and block radical chain reactions.

### 3.4. Antimicrobial Activity

#### 3.4.1. Antibacterial Activity

We evaluated the antimicrobial activity of extracts of* Citrus limon* by the method of diffusion in a solid medium. The activity was revealed on 11 bacterial strains Gram (+) and Gram (−). Then for each disk, we measured the diameters of zones of growth inhibition of bacterial cultures. The results of antibacterial screening extracts are shown in Table 3.

No zone of inhibition was observed in goshawks discs of lemon Zest after the end of incubation for most of the bacterial cultures listed above. These strains have a very high resistance against the action of this extract. For standard antibiotic (OFX), zones of inhibition ranged from 10 mm in* Proteus mirabilis* to 42 mm in* E. coli.* That antibiotic resistance was therefore seen in* E. coli, Citrobacter koseri, Streptococcus Group B, and Group D enterococci,* while other strains were sensitive to this antibiotic.* Pseudomonas aeruginosa* was resistant to rifampicin with an inhibition diameter of 22 mm.

When compared to the ZL extract inhibition, the FL extract presented significant values of inhibition for all bacterial strains. These values ranged between 16 and 32 mm.

The antibacterial test on *β* hemolytic* Streptococcus* showed growth inhibition for all extracts of both parts of lemon.

According to Massé et al. (2003) [52], sensitivity to Gram + bacteria is due to the inhibitory action on protein silybin synthesis and RNA. Furthermore, Pathak et al. (1991) [53] linked the sensitivity of bacteria to polyphenols to the inhibition of enzymes necessary for the production of energy in the bacterial cell or the change in the permeability of the cell and also to the inhibition of RNA synthesis.

In a study of the polyphenolic relationship, the antimicrobial potency of bacteria causing food spoilage, Lucera et al., (2012) [54] concluded that the sensitivity of microorganisms to polyphenols depends on itself and the structure of the polyphenol. However, knowledge of the action of antibiotics (action on Gram +) mechanisms can explain the sensitivity of the strains to these antibiotics.

#### 3.4.2. Antifungal Activity

The disk diffusion method allowed us to demonstrate the antifungal potency extracts of* Citrus limon* vis-à-vis the tested fungal strains.

The antifungal activity is indicated by the presence or the absence of mycelial growth. It results in a translucent halo around the sterile agar disc [55].

Only Nystatin antifungal drug used as a control at a dose of 100 *μ*g presented a zone of inhibition of growth of the strains, which confirms the validity of the method used.

No zone of inhibition was observed around discs impregnated with different extracts and none of the extracts inhibited the growth of these strains. This could be explained by the lack of substances with antifungal activity such as alkaloids [56]. These results indicate that ZL and FL extracts do not contain antifungal agents.

### 3.5. Antiproliferative Activity

**Figure 3:**
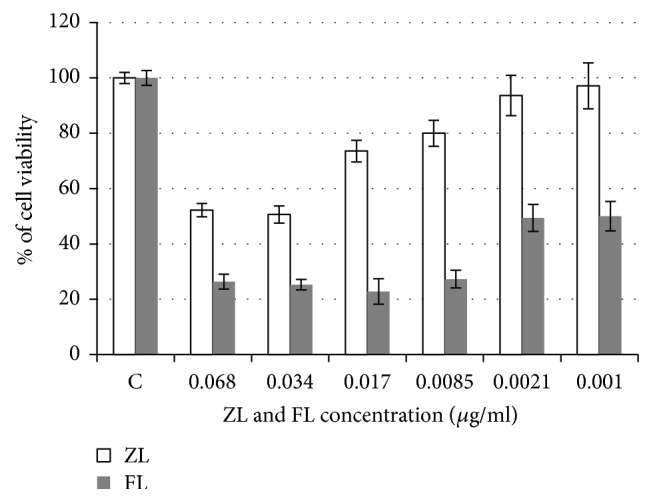
Effect of ZL and FL extracts of* Citrus limon* on proliferative activity of B95-8 cell line.

Cytotoxic effects on the line B95-8 were studied by MTT assay. Our results showed a cytotoxic effect of the extracts of the plant on line B95-8 (Figure 3); a dose-response was observed.

Our results showed that the extracts of* Citrus limon* have an inhibitory effect on the line B95-8 and this position is characterized by a remarkable increase in cytotoxicity as a function of increasing concentrations of the samples tested.

Cell proliferation was assessed by MTT assay using the B95-8 cells treated with varying concentrations of the extracts for 48 h. As shown in Figure 3, each sample inhibits cell proliferation in a dose-dependent manner. The proliferation of B95-8 cells was significantly reduced (*P* ≤ 0.001) by 50% after 48 h of exposure with 0.074 g/ml ZL or 0.0087 g/ml FL.

In a concentration of 0.015 g/ml of FL, only 20.11% of viable cells were present, while a concentration of 0.00087 had a low inhibitory power on B95-8 cells with a percentage of 95.44% of viability.

The strongest inhibitor power was observed at a concentration of 0.34 mg/ml for ZL (49.35% cell toxicity). Beyond these concentrations (0.015 and 0.034 mg/ml for ZL and FL, resp.), we observed a significant decrease in the inhibitory potency, and the effect of these extracts on the line B 95-8 was antagonistic.

### 3.6. Protein Damage Assay

**Figure 4:**
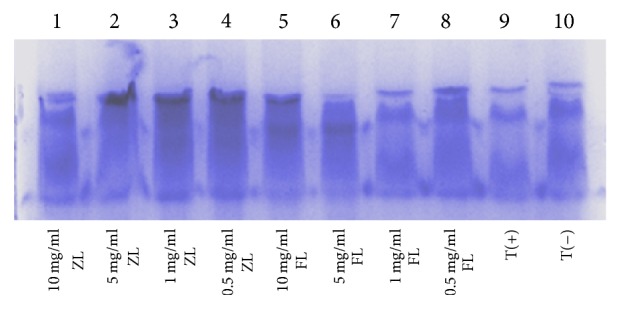
Effect of the ZL and FL* Citrus limon* extracts on protein damage.

Proteins are major targets for oxidants due to their high abundance in biological systems and high rate constants for the reaction of oxidants [57]. Previous scientific investigation has demonstrated that free radicals induced protein damage which plays a significant role in aging and pathological events. Electron leakage, metal-ion dependent reactions, and autooxidation of lipids and sugars have possibly led to radical-mediated damage to proteins [58]. Electrophoretic patterns of BSA after incubation with the Fe^3+^/H_2_O_2_/ascorbic acid system in the presence of samples were assayed with SDS–PAGE (Figure 4). In the current study, analysis of protein bands and quantified gel image showed the protective effect of both ZL and FL extracts against ROS attacks. At 1 mg/ml, extracts protected significantly BSA and remarkably restored the protein band intensity. This protective ability was mainly due the antioxidant activity of extracts. In fact, phenolic compounds are considered as major active components of the plant extracts responsible for the strong antioxidant capacity.

### 3.7. Inhibitory Effect of the* Citrus limon* Extracts on the Oxidative DNA Damage Caused by H_2_O_2_

**Figure 5:**
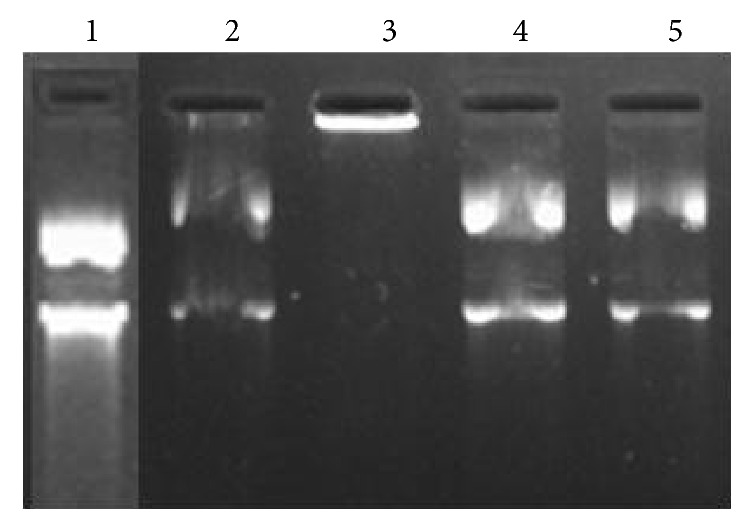
Inhibitory effect of the ZL and FL* Citrus limon* extracts on the oxidative DNA damage caused by H_2_O_2_.* Line 1*: untreated plasmid;* Line 2*: plasmid treated with 5 mg/ml ZL extract;* Line 3*: plasmid treated with 5 mg/ml of FL extract;* Line 4*: positive control (quercetin (10 mg/ml) + Rf Fenton);* Line 5*: negative control (plasmid + Rf Fenton).

The inhibitory effects of the Flesh and the peel extracted from lemon on oxidative DNA damage caused by H_2_O_2_ were investigated through* in vitro* DNA migration assay. According to Figure 5, a gel electrophoretogram of the FL and ZL effect on* in vitro *oxidative damage of plasmid DNA by hydroxyl radicals was generated through Fenton reaction between Fe^2+^ and H_2_O_2_. The plasmid DNA was mainly of the super-coiled form in the absence of Fe^2+^ and H_2_O_2_ (control). The addition of Fe^2+^ and H_2_O_2_ leads to the decrease of the DNA super-coiled form and conversion into the relaxed circular and linear form. The further fragmentation of linear form however decreased in the presence of FL and ZL. DNA migration assay is a sensitive biomarker of DNA damage. At concentrations of 5 mg/ml, we observed a significant dose-dependent decrease in DNA migration.

Flavonoids possess an ideal structure for trapping free radicals because they have a number of hydroxyls acting as hydrogen donors depicted as an important antioxidant [59]. This is shown in our results, since the lemon Flesh is richer in flavonoids than lemon Zest, which favors better DNA protection.

Numerous tumors and ROS-mediated signaling and genomic instability are marked by oxidative stress. It obviously contributes to the initiation and progression of cancer. About 80% of the DNA damage resulting in the development of cancer is caused by ROS such as hydrogen peroxide (H_2_O_2_), singlet oxygen (^1^O_2_), and hydroxyl radical (OH). Therefore, avoiding oxidative DNA damage induced by ROS is very important for cancer prevention [60].

## 4. Conclusion

In conclusion, the results of the present study indicate that the extracts from* Citrus limon* exhibit powerful antioxidant properties, expressed by its capacity to scavenge DPPH radicals and to reduce power, and the extracts reduce H_2_O_2_-induced DNA via its antioxidant activities. These antioxidant activities and inhibitory effects of the extracts on DNA and cell damage may further prove that* Citrus limon* is useful as a medicinal plant for cancer chemoprevention.

The results obtained show that* Citrus limon* extracts contain high enough levels of phenolic and flavonoid compounds. This is correlated with a remarkable antioxidant activity towards the reduction of iron, and a relatively high power against scavenging free radicals. So,* Citrus limon* extracts could be a promising antioxidant source for the prevention and/or treatment of oxidative stress-related diseases or as food additives.

## Figures and Tables

**Table 1:** Total phenolic (mg Eq Gallic Acid/g dry weight), flavonoids (mg Eq Quercetin/g dry weight), flavonols (mg Eq Rutin/g dry weight), and condensed tannins (mg Eq Catechin/g dry weight) of ZL and FL extracts of *Citrus Limon*.

	ZL	FL
Total phenol (mg Gallic acid Eq/g)	204.40 ± 09.62^*∗∗∗*^	105.55 ± 04.71
Total flavonoid (mg Quercetin Eq/g)	27.50 ± 06.88	56.16 ± 14.14^++^
Flavonols (mg Rutin Eq/g)	26.66 ± 07.07^*∗∗∗*^	09.16 ± 03.53
Condensed tannins (mg Catechin Eq/g)	138.33 ± 35.36^*∗∗∗*^	26.66 ± 18.92

^++^Correlation between FL and ZL difference was statistically significant (*P* < 0.01). ^*∗∗∗*^Correlation between ZL and FL difference was statistically significant (*P* < 0.001). The values are the mean of three determinations ± SD.

**Table 2:** Identification and analysis of the phenolic composition of ZL extracts using liquid chromatography high performance coupled with mass spectrometry (LC-MS/MS).

Pic	TR (min)	UV (nm)	[M-H]^−^	MS^2^	Structure
(1)	5.78	326	365.1446	263, 125, 142, 221, 302, 320	Caffeoyl N-Tryptophan
(2)	6.60	300 sh, 330	355.0666	147, 191, 209, 337	Fer-glc (acid Hydroxycinnamoyl-Oglucoside)
(3)	7.58	268, 338	593.1503	473, 353, 383, 503, 575	Vicenin 2
(4)	9.35	284, 334 sh	595.1659	287	Eriocitrin
(5)	10.02	256, 266, 350	593.1504	285, 151, 175, 199, 216, 241, 257	Kaempferol-3-O- rutinoside
(6)	11.09	263, 298 sh, 356	609.1819	301, 151, 178, 255, 271	Quercetin-3-rutinoside (Rutin)

**Table 3:** Diameter (mm) of inhibition zones of Microbial strains of *Citrus limon* extracts.

Bacterial strains	Diameter of inhibition (mm)	
	ZL	FL
*Escherichia coli *	0	16 ± 2^*∗∗∗*^
*Staphylococcus aureus*	0	30 ± 3^*∗∗∗*^
*Acinetobacter baumannii*	0	24 ± 2^*∗∗∗*^
*Proteus mirabilis*	0	19 ± 1.5^*∗∗∗*^
*Klebsiella pneumoniae*	0	22 ± 3.5^*∗∗∗*^
*Citrobacter koseri*	0	21 ± 1.6^*∗∗∗*^
*Salmonella enterica*	0	32 ± 1.9^*∗∗∗*^
*Pseudomonas aeruginosa*	0	22 ± 2.2^*∗∗∗*^
*Streptococcus agalactiae B*	32 ± 1.2	28 ± 2.6^*∗∗*^
*Streptococcus D*	21 ± 0.9	24 ± 1.9^*∗*^
*Enterococcus*	30 ± 3.1	31 ± 3.3

^*∗*^Correlation between FL and ZL inhibition was statistically significant (*P* < 0.05). ^*∗∗*^Correlation between FL and ZL inhibition was statistically significant (*P* < 0.01). ^*∗∗∗*^Correlation between FL and ZL inhibition was statistically significant (*P* < 0.001).
